# Pulmonary Embolism Despite Inferior Vena Cava Filter in a Polytrauma Patient: A Case Report

**DOI:** 10.1155/cro/1289499

**Published:** 2026-04-12

**Authors:** Gareth Ryan, Kurt Wilde, Robert Korley, Leslie Skeith, Prism Schneider

**Affiliations:** ^1^ Section of Orthopaedics, Department of Surgery, University of Calgary, Calgary, Alberta, Canada, ucalgary.ca; ^2^ Division of Hematology and Hematological Malignancies, Department of Medicine, University of Calgary, Calgary, Alberta, Canada, ucalgary.ca

**Keywords:** case report, Factor V Leiden, inferior vena cava filter, pulmonary embolism, trauma

## Abstract

**Case:**

A 39‐year‐old female with Factor V Leiden sustained multiple injuries following a motor‐vehicle collision. A prophylactic inferior vena cava filter (IVCF) was placed prior to fixation of her ipsilateral intertrochanteric and diaphyseal femur fractures the morning after admission. She developed a pulmonary embolism (PE) on postinjury Day 3. She underwent fixation of her ipsilateral tibial plateau and wrist on postinjury Day 6, IVCF removal on Day 12, and was subsequently discharged with 3 months of anticoagulation.

**Conclusion:**

PE can occur despite IVCF. Our understanding of the prevention and management of de novo PE is limited and should be the focus of future research.

## 1. Introduction

Venous thromboembolism (VTE) is a common complication in orthopedic patients. Despite thromboprophylaxis, up to 15.6% of patients with diaphyseal femur fractures develop a deep vein thrombosis (DVT) [1]. Pulmonary embolism (PE) is a serious complication that occurs in up to 2.2% of orthopedic trauma patients despite thromboprophylaxis [2]. Although PE is often thought to result from embolization of a DVT, up to 61% of PEs in trauma patients have no identified DVT on imaging [3]. The role of prophylactic inferior vena cava filters (IVCF) prior to surgery in high‐risk polytraumatized patients is controversial [4]. We present a case of an early postoperative PE in a polytraumatized patient despite preoperative IVCF placement.

The patient provided informed consent for her data to be published in this case report. This case report did not require research ethics board approval.

## 2. Case Presentation

A 40‐year‐old female was involved in a head‐on highway‐speed motor‐vehicle collision as a restrained driver. After a prolonged extrication, she was transported by air ambulance to our tertiary care center. She was hemodynamically stable upon arrival. She was found to have a comminuted left reverse oblique proximal femur fracture (AO/OTA 31A3.3), an ipsilateral diaphyseal femur fracture (AO/OTA 32B2), an ipsilateral tibial plateau fracture (AO/OTA 41C2), and a contralateral distal radius fracture (AO/OTA 23C2) (Figure 1). She additionally sustained a left C7 transverse process fracture, sternal body fracture with retrosternal hematoma. Her injury severity score was 24. All extremity fractures were closed and neurovascularly intact. She was admitted to the trauma service. Her past medical history was notable for elevated body mass index (BMI) of 38.1, heterozygous Factor V Leiden mutation, and hypothyroidism. She had no personal history of VTE, but her sister had suffered multiple unprovoked VTEs.

Figure 1Initial injury films in the emergency department. (a) Ipsilateral intertrochanteric and diaphyseal femur fracture, (b) Schatzker 6 tibial plateau fracture, and (c) comminuted distal radius fracture.

**Figure:**
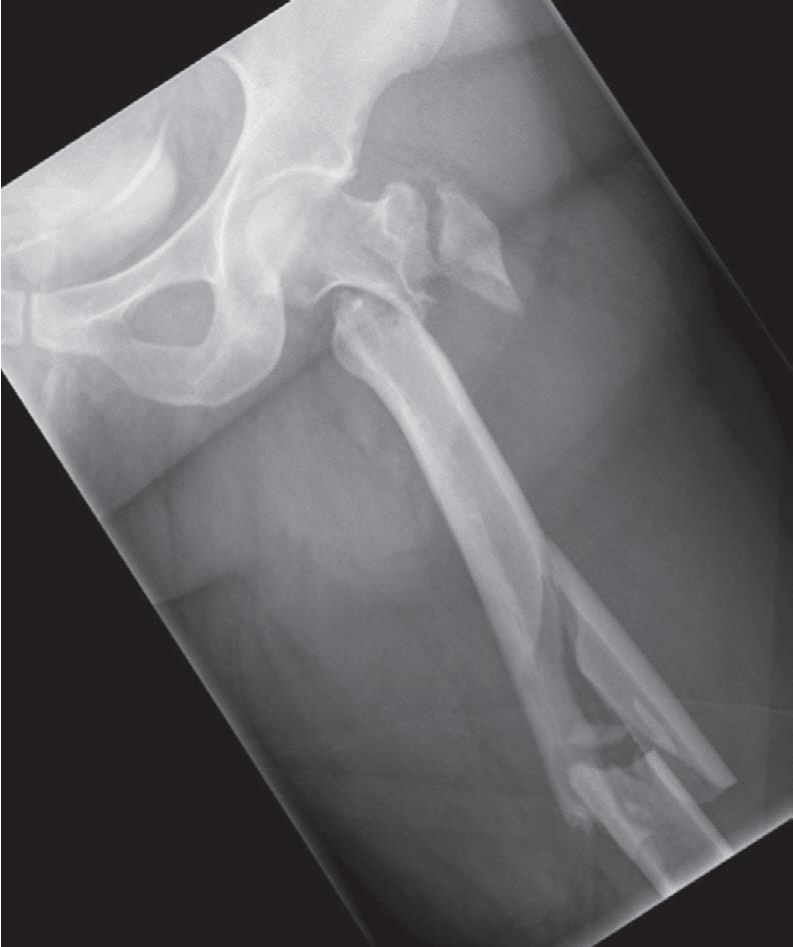
(a)

**Figure:**
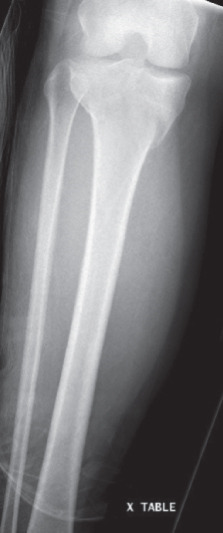
(b)

**Figure:**
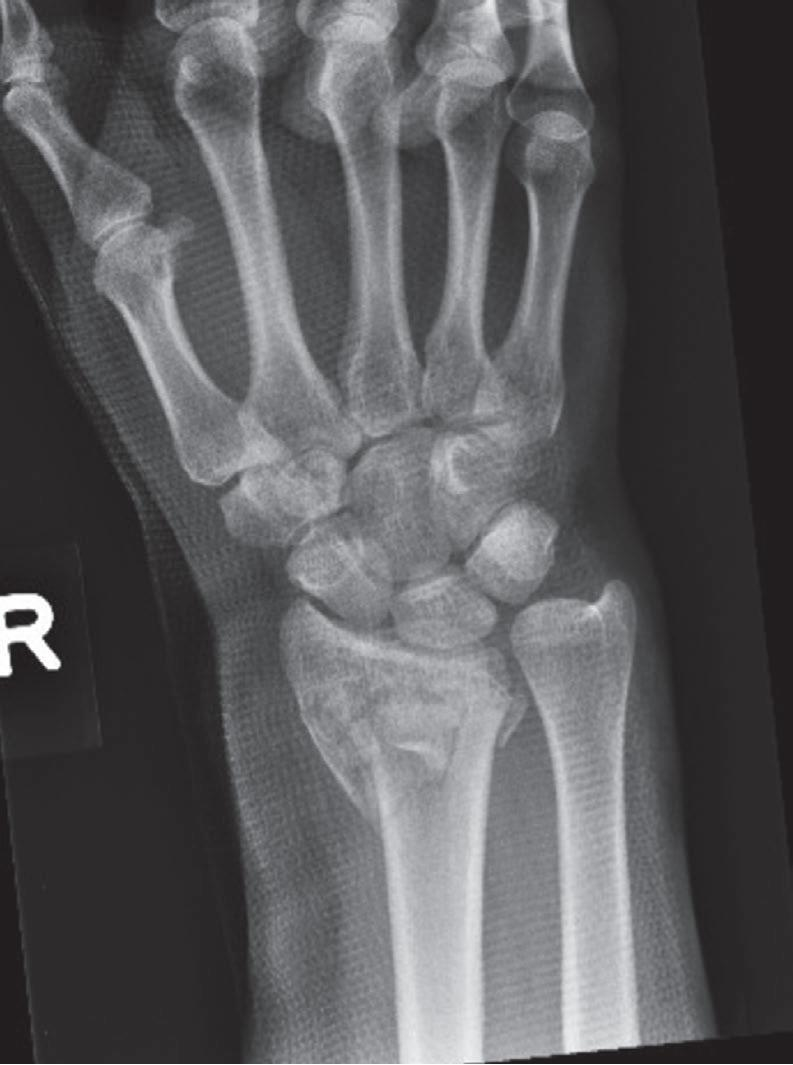
(c)

Given her increased risk of VTE secondary to her elevated BMI, thrombophilia, multisystem injuries, and the need for staged surgical management of her injuries, a multidisciplinary decision was made to place an IVCF preoperatively in collaboration with the trauma and hematology services. This was completed uneventfully by interventional radiology the morning of postinjury Day 1 prior to surgery (Celect Platinum Vena Cava Filter, COOK Medical, Bloomington, Indiana).

Fixation of her complex femur fracture was prioritized on postinjury Day 1, with a plan to manage her tibia and distal radius in a staged manner. She was taken to the operating room and placed under a general anesthetic. Given the complex pattern of her femur fracture, we elected to utilize two fixation constructs, with a dynamic hip screw with a trochanteric stabilization plate for her reverse obliquity fracture and a retrograde femoral nail for her diaphyseal fracture (Figure 2a). She was kept in a knee‐immobilizer rather than a temporizing knee‐spanning external fixator since her tibial plateau fracture was not grossly displaced. She received two units of blood intraoperatively and was hemodynamically stable throughout the procedure with no complications. She was brought to the postanesthetic care unit in stable condition and transferred back to the orthopedic unit later that evening. She was started on weight‐based prophylactic tinzaparin (75 units/kg/day), with a half dose given the following surgery and full dose beginning on postinjury Day 2.

Figure 2Radiographs at 6 months following surgery. (a) Ipsilateral intertrochanteric and femur fractures treated with dynamic hip screw and retrograde femoral nail, (b) Schatzker 6 tibial plateau treated with open reduction and medial and lateral plating, and (c) distal radius fracture treated with open reduction and dorsal and radial column plating.

**Figure:**
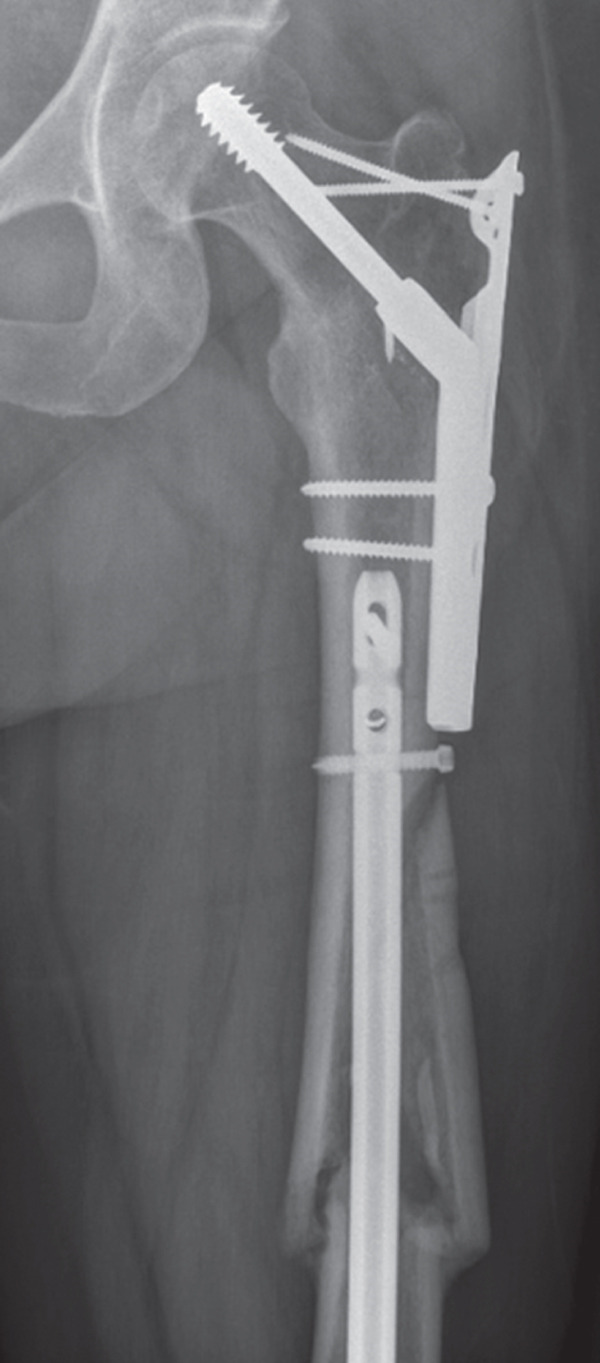
(a)

**Figure:**
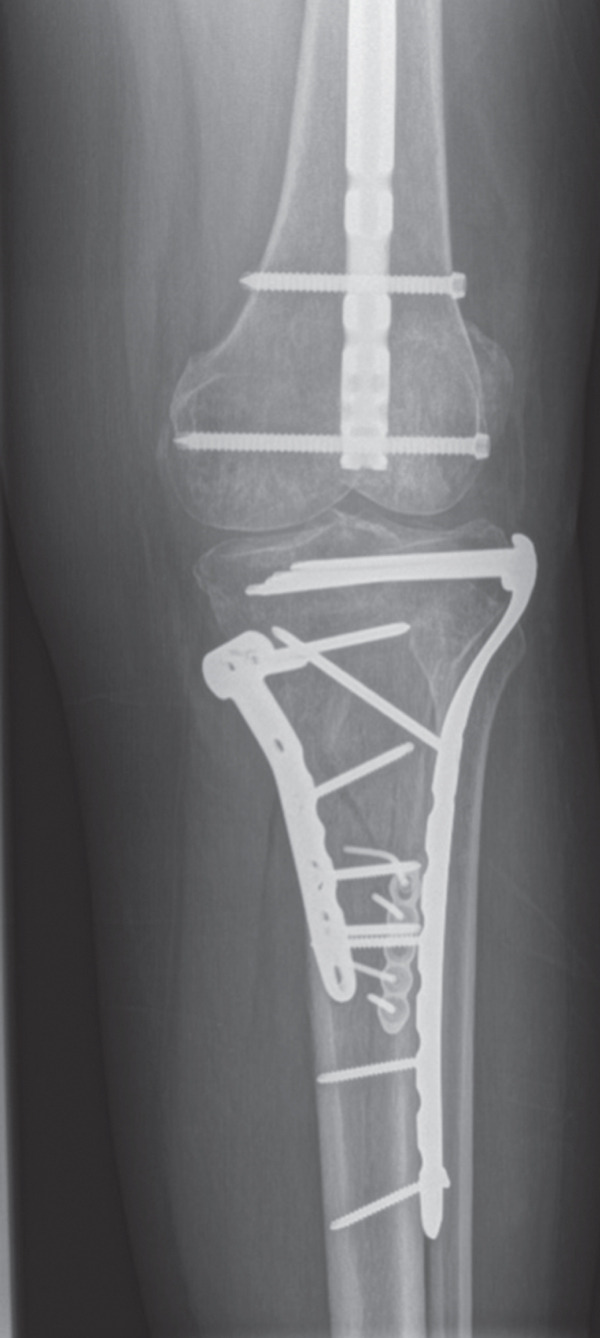
(b)

**Figure:**
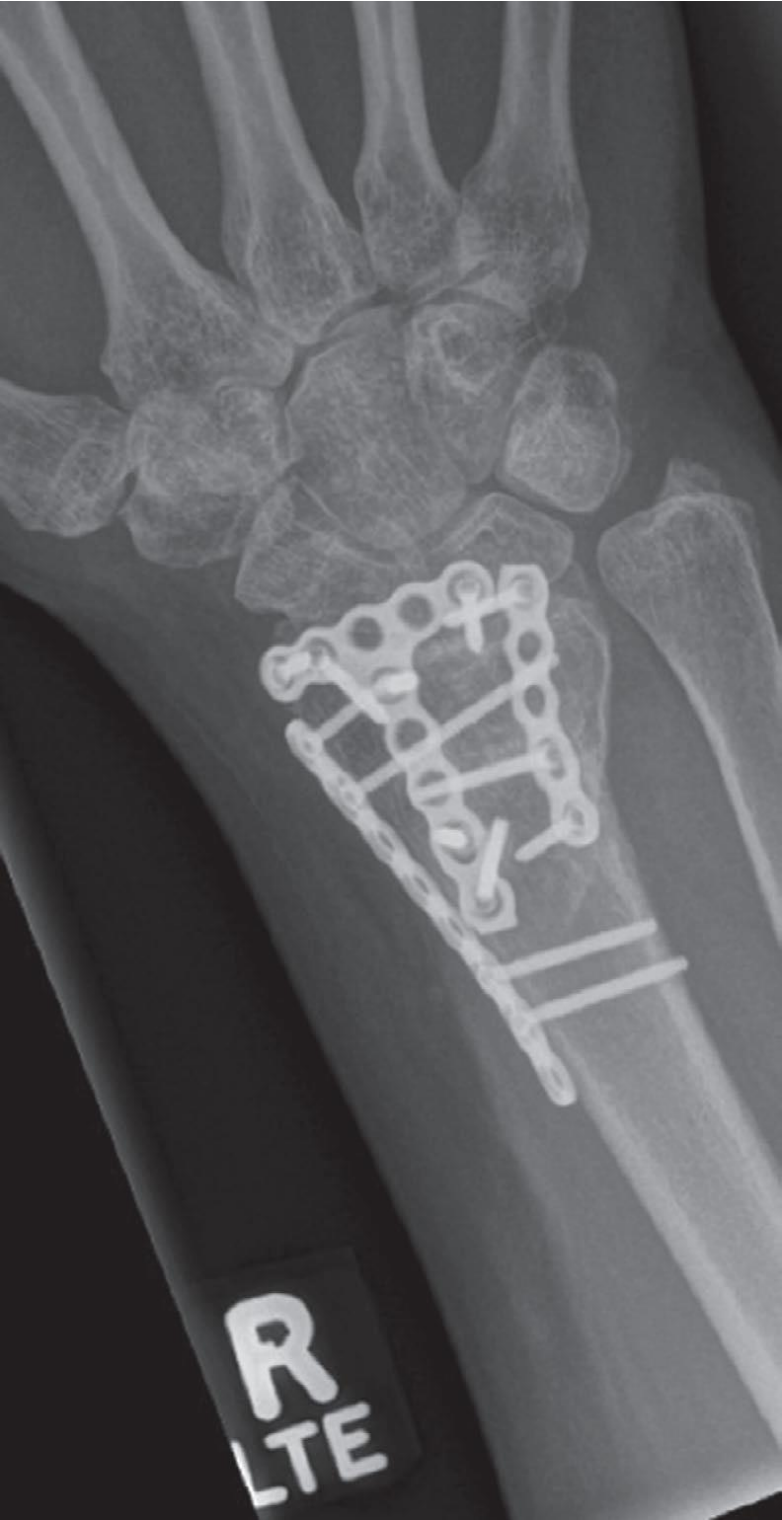
(c)

**Figure 3:**
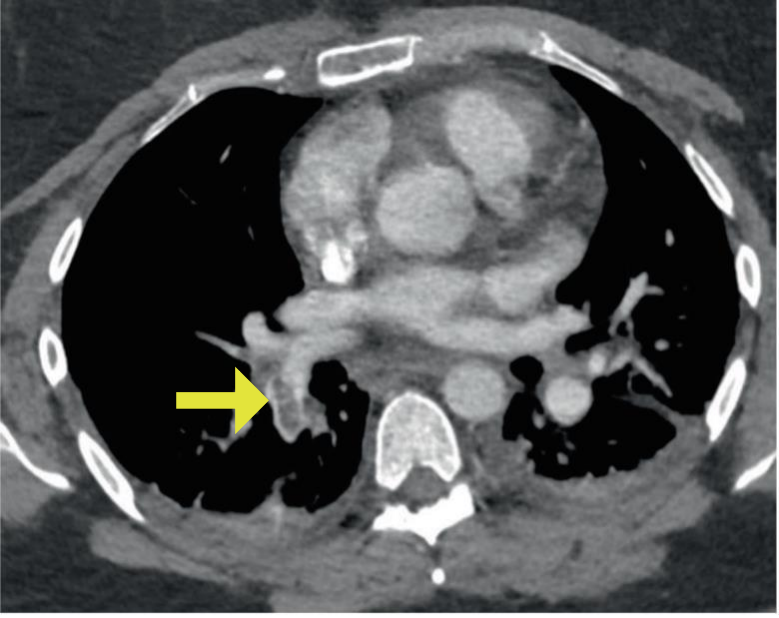
Axial computed tomography cut illustrating right lower lobar artery pulmonary embolism, as identified by yellow arrow.

**Figure 4:**
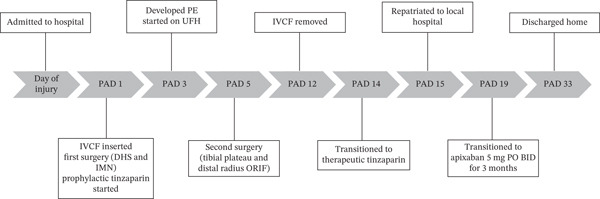
Timeline of events throughout admission and recovery. ∗Note: PAD, postadmission day; IVCF, inferior vena cava filter; DHS, dynamic hip screw; IMN, intramedullary nail; PE, pulmonary embolism; UFH, unfractionated heparin; ORIF, open reduction internal fixation; PO, by mouth; BID, two times per day.

She did well on postinjury Day 2. On postinjury Day 3, she was anemic with a hemoglobin of 69 and received two units of blood. She additionally endorsed dyspnea, pleuritic chest pain, and increasing oxygen requirements up to 5 L. She was otherwise vitally stable with a maximum heart rate of 92 and blood pressure straddling 110/60. PE and myocardial infarction were at the top of the differential diagnosis, with pneumothorax, myocarditis, pericarditis, tamponade, and dissection further down the list. An ECG and troponins were obtained, which were both normal. Computed tomography angiography of her chest was performed, which demonstrated an acute PE involving the right upper and lower lobar branches (Figure 3). There was no evidence of thrombus at the level of the IVCF and she had an echocardiogram that was unremarkable apart from turbulent pulmonary artery flow. The PE rapid response team was consulted, and she was started on intravenous unfractionated heparin (UFH) titrated to our hospital′s partial thromboplastin time‐based nomogram. She remained hemodynamically stable and her dyspnea resolved. She was taken back to the operating room on postinjury Day 5 for fixation of her left tibial plateau and right distal radius under a general anesthetic (Figure 2b,c). She received a further three units of blood intraoperatively. She tolerated her second surgery well and was extubated and transferred to the postanesthetic care unit before being brought to the floor. Her IVCF was removed on postinjury Day 12 and she was transitioned to weight‐based therapeutic dosing of tinzaparin (175 units/kg/day) on Day 13. She was repatriated to her local hospital on Day 15 for an additional 2 weeks of inpatient rehabilitation. She was transitioned to a 3‐month course of apixaban 5 mg twice daily on postinjury Day 19. The timeline of key events during her admission is summarized in Figure 4.

Notably, the patient was enrolled in a research study evaluating perioperative coagulopathy using serial thrombelastography (TEG 6S, Haemonetics, Braintree, Massachusettes). Using a maximal amplitude threshold of ≥ 65 mm, she demonstrated a hypercoagulable state from postoperative Day 3 until 3 months despite therapeutic anticoagulation [5, 6]. These data were collected solely for research purposes, as thrombelastography is currently being investigated as a tool to define hypercoagulability, but is not being used to direct clinical care by the orthopedics and trauma services at our center.

She remained nonweightbearing on the left leg until 8 weeks with the assistance of a forearm walker and wheelchair. She was then permitted to gradually increased to weightbearing‐as‐tolerated from 8 to 12 weeks postoperatively. She was seen in the outpatient thrombosis clinic 3 months following her PE at which point her apixaban was discontinued given the provoked nature of her PE. She ultimately made a full recovery from her PE and continues to work with physiotherapy for her orthopedic injuries. She was weaning off of her cane 1 year out from her injury.

## 3. Discussion

The majority of orthopedic trauma patients demonstrate a profound hypercoagulable state from postoperative Day 3 onwards, with persistent hypercoagulability in over 50% of patients with hip fractures at 6 weeks [7]. Despite most patients demonstrating hypercoagulability, the incidence of posttraumatic PE is as low as 0.3%, partly due to widespread use of thromboprophylaxis [8]. This case highlights several important concepts in the management of perioperative coagulopathy in trauma patients.

The pathophysiology of PE is complex. Historically, thrombosis of the pulmonary vessels was thought to occur solely from embolization of a DVT. In the setting of trauma, 61%–86% of pulmonary thromboses occur without an antecedent DVT, more accurately referred to as de novo PE, in situ PE, or pulmonary arterial thrombosis [3, 8–14]. In contrast, only 8% of PEs in nontrauma patients are de novo and often seen in the setting of malignancy [15]. The high frequency of de novo PE in trauma patients suggests that the pathophysiology of clot formation in the setting of trauma may be distinct or multifactorial. Alternate explanations for the absence of DVT have been proposed, including underdiagnosed DVT in the pelvic or other vessels and complete embolization. Hemodynamic instability on admission, major chest injury with an abbreviated injury score of three or higher, major venous injury, liver injury, and younger age have been identified as independent risk factors for development of de novo PE [3, 11, 16].

Insertion of an IVCF is intended to prevent embolization of a DVT into the lungs; however, patients can still develop a PE secondary to clot breakthrough, collateral flow, IVCF thrombosis, or de novo PE [17]. This is especially relevant in trauma patients given the high incidence of de novo PE or potential blood vessel injury and related thrombus formation in other locations. Although insertion of IVCF in the setting of acute DVT and inability to therapeutically anticoagulate is an accepted indication, the use of prophylactic IVCF is controversial based on limited data [18]. In patients with a history of VTE who are undergoing elective arthroplasty, prophylactic placement of an IVCF may reduce the risk of PE [19]. In the setting of trauma, observational studies have shown that IVCF insertion is associated with increased risk of VTE; however, there is considerable treatment bias [20–22]. Although observational studies may suggest possible benefit [23], randomized‐controlled trial (RCT) data have shown no benefit of prophylactic IVCFs [23, 24]. Only three RCTs have been performed evaluating prophylactic use of IVCFs in trauma patients, with two of the trials focusing on feasibility [25–27]. The third trial, which is the only full‐size RCT to date, demonstrated no benefit of prophylactic IVCF, with an incidence of symptomatic PE of 13.9% and 14.4% in the IVCF and control groups, respectively [27]. Additional downsides of IVCF include vessel injury, bleeding, and migration, which may complicate a trauma patient′s hospital course. Approximately 6% of patients experience acute complications following IVCF insertion, with unsuccessful filter retrieval in up to 18% [28–30]. The risk of filter‐related complications is likely considerably increased in trauma patients, where loss to follow‐up is common. The overall quality of evidence surrounding prophylactic use of IVCF in trauma patients is poor, making decisions surrounding insertion and subsequent removal challenging, especially as our understanding of de novo PE improves.

Although up to 80% of all VTEs occur beyond 1 week from injury, 21% of PEs occur in the first 48 h and 37% occur within 4 days [9, 10, 31]. Early PE (within 72 h) is more common in patients with multiple lower extremity fractures and in patients who undergo definitive fixation within 48 h [32, 33]. Counterintuitively, early PE has been shown to be associated with lower injury severity scores, regional/spinal anesthesia, and shorter time to initiation of thromboprophylaxis [32, 33]. As such, it is important to have a low threshold for ruling out PE in trauma patients at any timepoint, regardless of the magnitude of their injuries.

Another important component of this case is the presence of a thrombophilia. Factor V Leiden is a mild and common thrombophilia in 5% of the general and orthopedic surgery population [34]. Factor V is responsible for conversion of prothrombin to thrombin and is regulated by protein C. Factor V Leiden results in unregulated thrombus formation due to the absence of the protein C binding site. Although individuals with Factor V Leiden are at increased baseline and postoperative risk of VTE (odds ratio 18.6–24.3), perioperative thromboprophylaxis guidelines are identical to the general population [35, 36]. As such, thrombophilia in isolation is not an indication for prophylactic IVCF, but should be considered in conjunction with other VTE risk factors in multidisciplinary consultation.

In summary, this case highlights several important concepts in perioperative thromboprophylaxis. Trauma patients likely demonstrate a unique coagulopathy, with a propensity towards early postinjury de novo PE formation without prior DVT. Although IVCF is an option, the evidence surrounding its prophylactic use is limited. The high rate of de novo PE in trauma patients should be considered when determining the role of prophylactic IVCF. Further research and guidelines surrounding perioperative thromboprophylaxis for patients with increased baseline risk of VTE are needed. The focus of our laboratory is the use of thrombelastography as a patient‐specific metric of coagulopathy, which has shown considerable promise in trauma patients to date. This technology may assist in early identification of patients at high risk of VTE, thereby reducing morbidity and mortality.

## Funding

No funding was received for this manuscript.

## Conflicts of Interest

The authors declare no conflicts of interest.

## Data Availability

The data that support the findings of this study are available on request from the corresponding author. The data are not publicly available due to privacy or ethical restrictions.
